# Integrating bioinformatics and machine learning to discover sumoylation associated signatures in sepsis

**DOI:** 10.1038/s41598-025-96956-x

**Published:** 2025-04-24

**Authors:** Xue Teng, Qi Wang, Jinling Ma, Dongmei Li

**Affiliations:** 1 Department of Anesthesiology, Heilongjiang Provincial Hospital, Harbin, Heilongjiang China; 2 Department of Colorectal Surgery, Harbin Medical University Cancer Hospital, Harbin, Heilongjiang China; 3 Department of Intensive Care Medicine, Heilongjiang Provincial Hospital, Harbin, Heilongjiang China; 4 Department of Anesthesiology, The Second Affiliated Hospital of Harbin Medical University, Harbin, Heilongjiang China; 5 The Key Laboratory of Anesthesiology and Intensive Care Research of Heilongjiang Province, Harbin, Heilongjiang China

**Keywords:** Sepsis, SUMOylation, Machine learning, Hub genes, Immune cell infiltration, Computational biology and bioinformatics, Biomarkers, Pathogenesis

## Abstract

Small Ubiquitin-like MOdifier-mediated modification (SUMOylation) is associated with sepsis; however, its molecular mechanism remains unclear. Herein, hub genes and regulatory mechanisms in sepsis was investigated. The GSE65682 and GSE95233 datasets were extracted from public databases. Differential analysis and Weighted Gene Co-expression Network Analysis (WGCNA) were conducted in GSE65682 to identify differentially expressed genes (DEGs) and key module genes. Candidate genes were derived by intersecting with SUMOylation-related genes (SUMO-RGs). The Least Absolute Shrinkage and Selection Operator (LASSO) and Support Vector Machine-Recursive Feature Elimination (SVM-RFE) were utilized to identify significant feature genes. The convergence of those genes was utilized for diagnostic assessment and expression validation. Hub genes were defined as those exhibiting an area under the curve (AUC) greater than 0.7, significant gene expression, and a consistent trend. Localization and functional analyses of hub genes were conducted to enhance the understanding of these genes. Immune analysis, regulatory network construction, and drug prediction were performed. Six hub genes were identified: RORA, L3MBTL2, PHC1, RPA1, CHD3, and RANGAP1. These genes possessed considerable diagnostic significance for sepsis and were also markedly downregulated in the condition. Hub genes were predominantly enriched in the ribosome pathway and exhibited a strong correlation with differential immune cells. Activated CD8 + T cells exhibited a positive correlation with RORA. Based on the predicted and established regulatory network, AC004687.1 was observed to modulate PHC1 expression via hsa-miR- 142 - 5p. A total of six hub genes (RORA, L3MBTL2, PHC1, RPA1, CHD3, and RANGAP1) associated with SUMOylation was identified in sepsis in the current study. The findings are likely to aid in the differentiation between control and disease states, offering substantiation for the diagnosis of sepsis.

**Supplementary Information:**

The online version contains supplementary material available at 10.1038/s41598-025-96956-x.

## Introduction

Sepsis is a life-threatening systemic inflammatory syndrome caused by bacterial infection, resulting in multiple organ failure^1^. An imbalance of excessive inflammation and anti-inflammatory response, resulting from chronic immune dysfunction, has been recognized as the principal cause of organ dysfunction and mortality in sepsis. Globally, it is estimated that there are 48.9 million cases of sepsis each year, leading to 11 million fatalities^2^. A meta-analysis encompassing 170 global studies revealed a 90-day mortality rate of 32.24% for patients with sepsis^3^. Despite advancements in the prompt administration of antibiotics, fluid resuscitation, and multi-organ support, the mortality rate of sepsis has gradually diminished; nevertheless, the prognosis for patients with sepsis remains unfavorable^4,5^. The primary cause of sepsis is an exaggerated response to external injury factors, leading to remote organ damage^6^. Consequently, a comprehensive understanding of the pathogenesis and the identification of a novel biomarker for sepsis diagnosis is urgently required to enhance treatment efficacy.

Small ubiquitin-like modifier (SUMOylation) is a significant dynamic process of post-translational modification (PTM)^7^. The localization and function of a target protein can be modulated through covalent modification of the substrate protein. SUMO participates in numerous cellular processes, including protein stability and metabolism, nucleoplasmic transport, transcriptional regulation, apoptosis, inflammation, and cell cycle progression^8,9^, along with the regulation of mitochondrial division, ion channels, and biological rhythms^7^. Furthermore, SUMOylation participates in numerous cellular inflammatory processes^10^. Immune cells, including macrophages and dendritic cells, are integral to all phases of sepsis and influence immune homeostasis and inflammatory responses^11–13^. Furthermore, exploring the potential of SUMOylation as a critical factor for sepsis diagnosis may yield significant insights into pathogenesis and enhance outcomes. Consequently, it is essential to develop a sepsis diagnosis model utilizing SUMOylation-related genes to identify a gene signature. This study identified key genes via the Gene Expression Omnibus (GEO) in conjunction with SUMOylation-related genes, and systematically analyzed their association with immune infiltration and molecular regulatory networks. The resultant findings are highly likely to offer critical insights into the diagnosis and treatment of sepsis.

## Materials & methods

### Data extraction

The GSE65682 dataset was obtained from the GEO database (https://www.ncbi.nlm.nih.gov/gds) as a training set, consisting of 802 blood samples (42 control and 760 sepsis). The GSE95233 dataset, utilized as a validation set, was sourced from the GEO database and comprises 102 blood samples (22 control and 80 sepsis). In addition, the datasets GSE28750, GSE134347, also from the GEO database, containing 58 (20 control and 38 sepsis) and 238 (82 control and 156 sepsis) blood samples each were used to validate the expression of the core genes. A total of 187 SUMOylation-related genes (SUMO-RGs) were obtained from MSigDB using “sumoylation” as the search term. The main elements of the study was shown in the flowchart Supplementary Figure S1.

### Difference analysis

The differentially expressed genes (DEGs) in the GSE65682 dataset (sepsis vs. control) were screened using the limma package with |log_2_ (Fold Change) FC| > 0.5 & a *p*< 0.05^14^.

### Weighted gene Co-expression network analysis (WGCNA)

The GSE65682 dataset was analyzed using the WGCNA package^15^to identify modules that were significantly correlated with sepsis. A cluster analysis was conducted to identify and remove any anomalous samples adversely affecting precision. Secondly, a scale-free R^2^ threshold of 0.85 was attained, and the optimal soft threshold (β) was established based on the trend of mean connectivity approaching 0. The network was subsequently constructed utilizing this optimal soft threshold, with the heterogeneity coefficient computed. The dynamic tree cutting algorithm was utilized to obtain co-expression modules, necessitating a minimum of 200 genes per module. Finally, the module exhibiting the highest Pearson correlation analysis of sepsis was designated as the key module, and the genes contained within this module were classified as key module genes.

### Acquisition and enrichment analysis of candidate genes

Candidate genes were identified by synthesizing the intersection of differentially expressed genes (DEGs), key module genes, and SUMO-related genes (SUMO-RGs). Gene Ontology (GO) and Kyoto Encyclopedia of Genes and Genomes (KEGG)^16–18^enrichment analyses were performed utilizing the clusterProfiler package^19^, with a significance threshold established at *p* < 0.05 to investigate the functions and pathways related to these genes. The enriched pathways were then visualized using the tree map package. Furthermore, to investigate possible interactions among these candidate genes, a protein-protein interaction (PPI) network was constructed using the STRING database (http://string-db.org/).

### Machine learning

The Least Absolute Shrinkage and Selection Operator (LASSO), through its integration of L1 regularization, effectively fulfills both feature selection and model training roles. This approach is especially beneficial for high-dimensional data, as it efficiently eliminates coefficients of non-essential features, thereby emphasizing the important ones. The relatively low computational complexity enables effective management of large datasets. Furthermore, the Support Vector Machine-Recursive Feature Elimination (SVM-RFE) method iteratively eliminates less significant features by leveraging SVM weight vectors to evaluate feature importance, which theoretically results in more reliable and accurate feature selection results. Thus, the intrinsic capability of SVM in managing high-dimensional data, along with the stepwise feature reduction provided by RFE, facilitated the identification of an optimal feature subset. Two machine learning algorithms were utilized to identify hub genes, relying on the training set for analysis. Initially, LASSO regression analysis was performed on the candidate genes utilizing the “glmnet” package^20^, leading to the identification of feature genes. A 10-fold cross-validation was used to determine the optimal value of λ by minimising the Mean Squared Error (MSE) (lambda.min). Subsequently, SVM-RFE was employed using the “e1071” package^21^ to assess the relative importance and ranking of each gene among the candidates, identifying the feature gene with the lowest error rate. In this, the optimal combination of parameters was determined by 10-fold cross-validation, and parallel computing was used to accelerate the feature selection process and selected the most appropriate number of features based on a plot of generalisation error versus the number of features. Finally, the intersection of the feature genes identified by both algorithms was achieved, leading to the identification of the candidate hub genes.

### Diagnostic analysis and expression validation

To evaluate the distinguishing significance of potential hub genes between sepsis and control in the training set, the pROC package^22^ was utilized to generate the receiver operating characteristic (ROC) curves for each gene in both the training and validation sets. A gene exhibiting an area under the curve (AUC) exceeding 0.7 was deemed to possess a relatively robust diagnostic performance. Furthermore, an analysis of gene expression was conducted in both the training and validation sets to ensure consistency. Finally, the genes that demonstrated notable expression and stable patterns were identified as hub genes.

### Gene set enrichment analysis (GSEA) analysis

A correlation analysis was conducted using the “Spearman” method with the psych package to investigate the potential biological processes and signaling pathways associated with the identified hub genes^23^. The outcomes were organized based on the correlation coefficient. The clusterProfiler package was employed to conduct GSEA enrichment analysis using c2.cp.kegg.v2023.1.Hs.symbols as the background gene set (|NES| > 1 & *p*.adjust < 0.05).

### Immune analysis

To investigate the immune microenvironment in sepsis, ssGSEA was utilized on the training set to assess the abundance of 28 immune cells across all samples. A Wilcoxon test was then performed to analyze the differences between the sepsis and control groups. Subsequently, Spearman analysis was employed to explore the relationship between the differentially infiltrating immune cells and hub genes.

### Localization and interaction analysis

The subcellular localization of hub genes was predicted utilizing the UniProt (https://www.uniprot.org/)^24^ and HPA databases (https://www.proteinatlas.org/)^25^. Furthermore, the localization of the hub genes on chromosomes was illustrated through the use of the “RCircos” package^26^, facilitating a clear representation of the distribution of hub genes across the chromosomal landscape. Finally, the gene-gene interaction (GGI) network was constructed using the GeneMANIA database (https://genemania.org/), allowing for an exploration of the interactions between hub genes and other genes.

### Construction of regulatory network

The hub genes that are regulated by miRNAs were identified through the use of miRDB (https://mirdb.org/) and Starbase (https://starbase.info/). The miRNAs identified by both databases were analyzed and cross-referenced. The Starbase (https://starbase.sysu.edu.cn/index.php) was subsequently utilized to predict and identify lncRNAs that regulated the leading three miRNAs. Finally, the lncRNA-miRNA-mRNA network was visualized using the Cytoscape software^27^, facilitating the depiction of regulatory interactions among the lncRNAs, miRNAs, and mRNAs.

### Drug prediction

To investigate potential therapeutic drugs, the CTD database (https://ctdbase.org/) was employed to forecast small molecules that target the hub genes. A network depicting gene-drug interactions was created using Cytoscape software^27^.

### Statistical analysis

The data was processed and analyzed utilizing R software, employing the Wilcoxon rank-sum test to assess differences. A significance threshold of *P* < 0.05 was utilized to determine the presence of statistically significant differences.

## Results

### Identification and analysis of DEGs and co-expression modules in sepsis

**Fig. 1:**
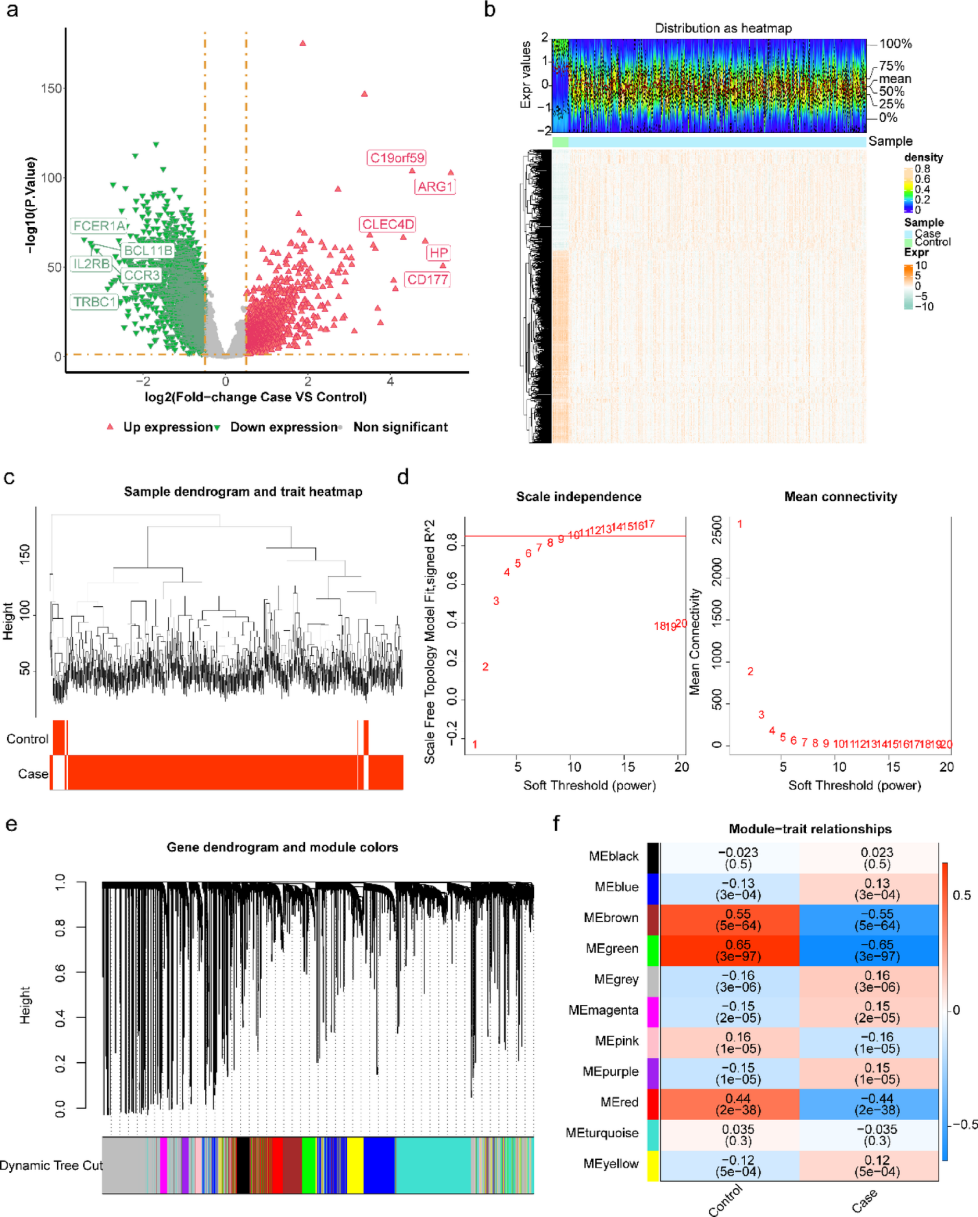
Integrated analysis of differential gene expression and clustering in GSE65682 dataset. (**a**) Volcano plot showing DEGs between the case and control groups in the GSE65682 dataset. Red triangles represent upregulated DEGs, green triangles represent downregulated DEGs, and grey indicates genes that are not statistically significant. (**b**) Heatmap displaying DEGs between the case and control groups in the GSE65682 dataset. (**c**) Sample clustering dendrogram with branches representing samples and the y-axis indicating hierarchical clustering height. (**d**) Soft threshold and scale-free topology fitting index. (**e**) Gene hierarchical structure tree clustering diagram. (**f**) Heatmap showing the correlation between modules and traits (case and control) in the GSE65682 dataset.

In the GSE65682 dataset, a total of 3,889 DEGs (1,268 up-regulated and 2,631 down-regulated) were identified in sepsis compared to the control group (Fig. 1a, b). Figure 1c displays the cluster analysis, revealing no outliers among the samples. The optimal β was established at 12 when R^2^ neared the threshold of 0.85, while the mean connectivity approached 0 (Fig. 1 d, e). A total of eleven co-expression modules were identified through similarity analysis and module merging. The heat map illustrates the correlation between sepsis and modules, with the green module (*r* = − 0.65, *p* < 0.001) consisting of 638 genes exhibiting the strongest association with sepsis (Fig. 1f).

### Functional analysis and protein-protein interactions of candidate genes in sepsis

**Fig. 2:**
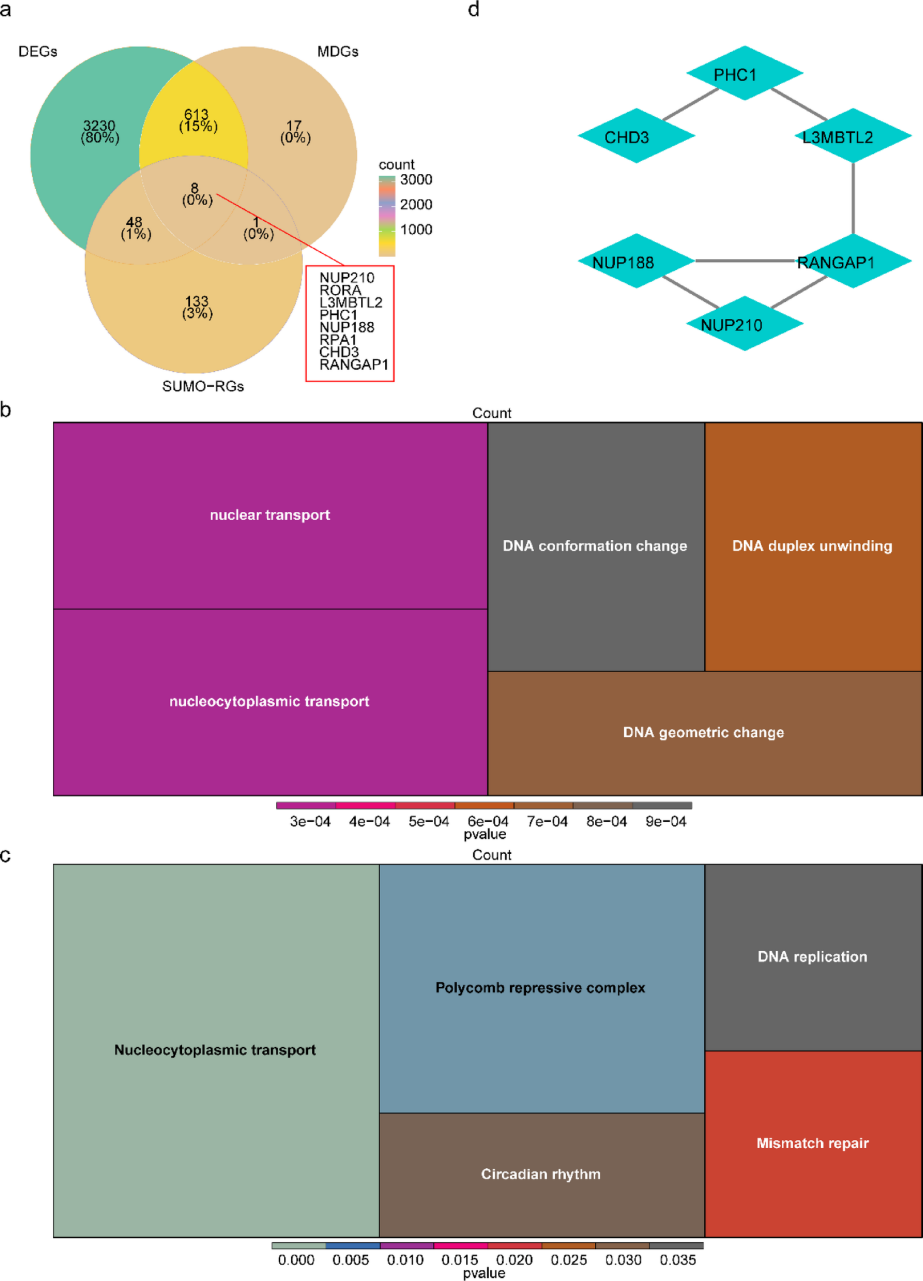
Analysis of function and protein-protein interactions of candidate genes in sepsis. (**a)** Venn diagram of the intersection of DEGs, key module genes and SUMO-RGs, **(b)** GO enrichment analysis of candidate genes, with each square representing a pathway, the size of the square indicating the number of genes in the pathway, and color indicating significance. **(c)** KEGG enrichment analysis of candidate genes, with each square representing a pathway, the size of the square indicating the number of genes in the pathway, and color indicating significance. **(d)** PPI network of candidate genes.

Eight candidate genes were obtained through integrating the intersection of DEGs, key module genes and SUMO-RGs (Fig. 2a). Subsequently, GO and KEGG analyses were conducted to predict the functionality of candidate gene enrichment. Based on the GO, candidate genes were found to be predominantly enriched in nuclear transport, nucleocytoplasmic transport, DNA conformation alteration, DNA duplex unwinding, and DNA geometric modification (Fig. 2b). Conversely, the candidate genes were found to be primarily associated with nucleocytoplasmic transport, polycomb repressive complex, circadian rhythm, DNA replication, and mismatch repair as per KEGG analyses (Fig. 2c). Furthermore, PPI networks were established to investigate the interactions among the proteins encoded by these candidate genes. The highest frequency of interactions was noted between RANGAP1 and NUP188, NUP210, or L3MBTL2 (Fig. 2d). Among them, The centrality of these six genes was shown in Supplementary Table S1, with degree values of 2 for L3MBTL2, PHC1 and RANGAP1.

### Identification of candidate hub genes in sepsis by machine learning algorithms

**Fig. 3:**
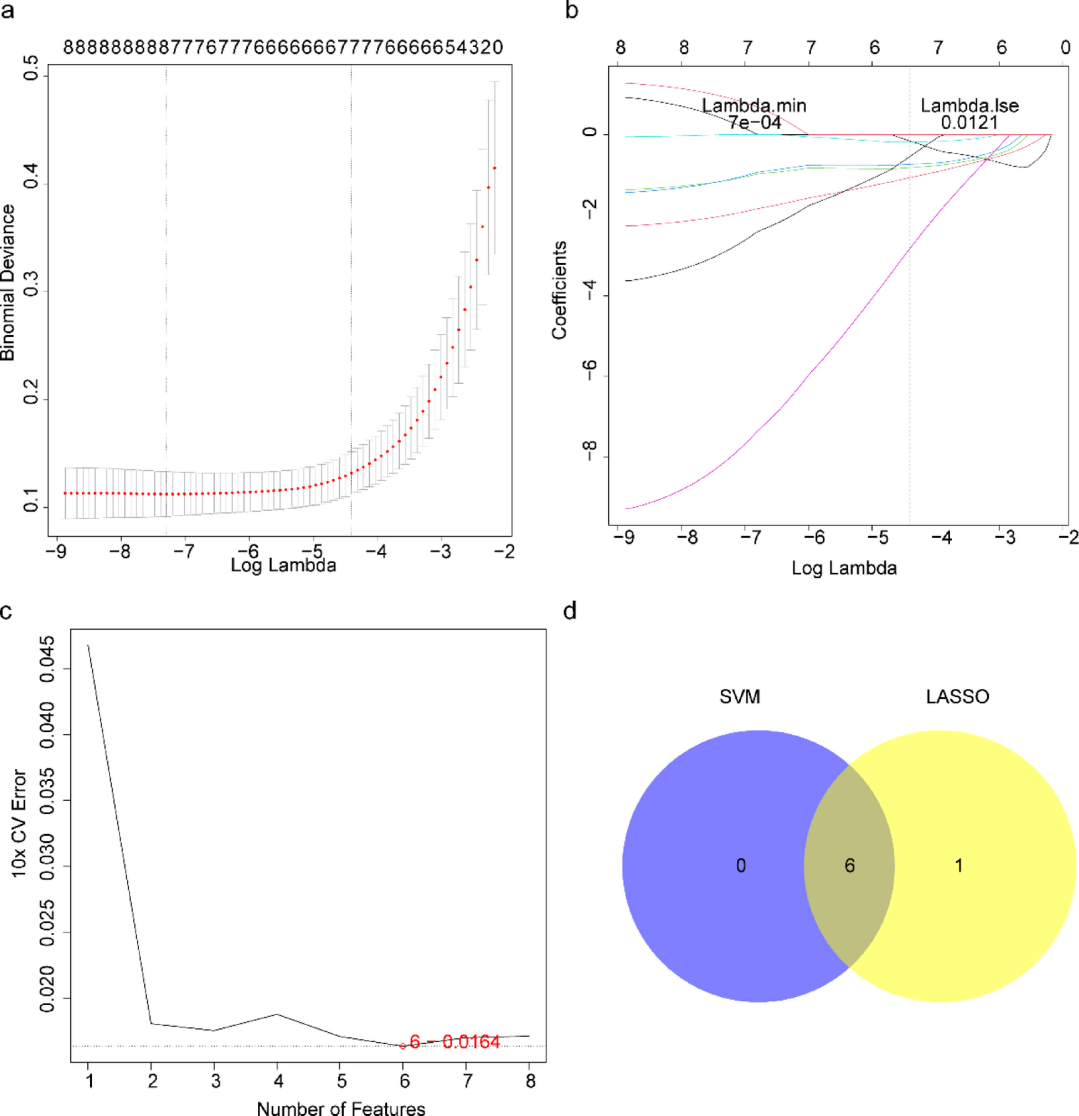
Identification of candidate hub genes using LASSO regression analysis and SVM-RFE. (**a)** Cross-validation curve of LASSO regression analysis. (**b)** LASSO coefficient path plot. (**c)** Feature gene selection by the SVM-RFE algorithm. (**d)** Venn diagram showing overlapping feature genes selected by LASSO and SVM-RFE.

The feature genes were individually selected using two distinct machine learning algorithms. Seven candidates were identified through the LASSO algorithm: NUP210, RORA, L3MBTL2, PHC1, RPA1, CHD3, and RANGAP1 (Fig. 3a, b). Furthermore, six feature genes were identified using the SVM-RFE algorithm, comprising related orphan receptor A (RORA), lethal (3) malignant brain tumor-like protein 2 (L3MBTL2), replication protein A1 (RPA1), chromodomain-helicase-DNA-binding protein 3 (CHD3), and RanGTPase activating protein 1 (RANGAP1). These six genes demonstrated robust diagnostic efficacy with consistent and significant expression patterns across both datasets, thus they were classified as key genes (Fig. 3c). Finally, the convergence of two machine learning algorithms yielded six candidate hub genes for further analysis: RORA, L3MBTL2, PHC1, RPA1, CHD3, and RANGAP1 (Fig. 3d).

### Hub genes and their potential interactions in sepsis

**Fig. 4:**
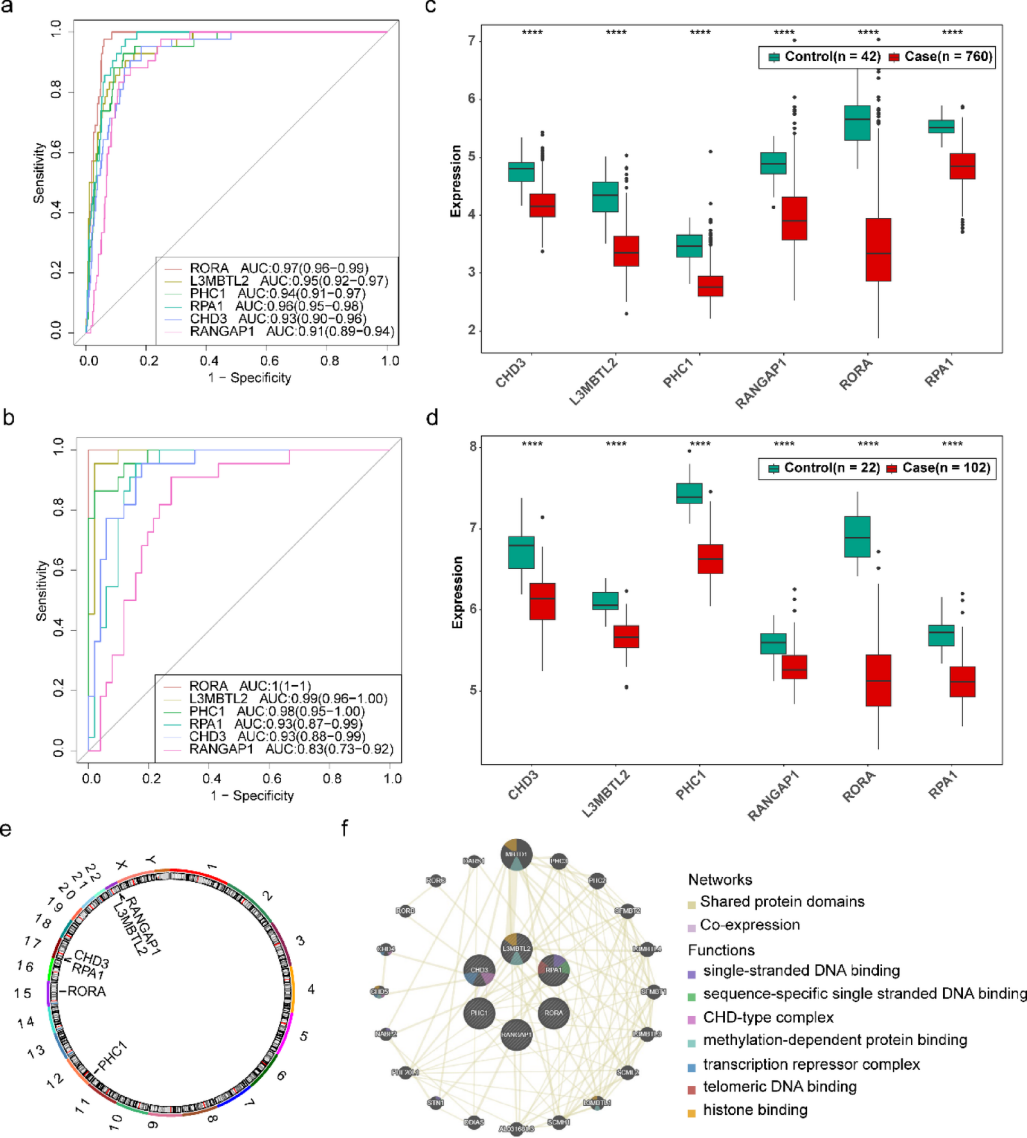
Identification and characterization of hub genes in sepsis. (**a)** ROC curve of candidate hub genes in the training set. (**b)** ROC curve of candidate hub genes in the verification set. (**c)** Expression of candidate hub genes in the training set. (**d)** Expression of candidate hub genes in the verification set. (**e)** Chromosome localization of hub genes. (**f)** The GeneMANIA Network. * *p* < 0.05; ** *p* < 0.01; *** *p* < 0.001; **** *p* < 0.0001.

ROC curves were generated to assess the diagnostic potential of candidate hub genes for sepsis. The AUC values of six genes in both the training and validation sets exceeded the threshold of 0.7, indicating their efficacy in distinguishing sepsis from control (Fig. 4a, b). Expression validation was performed to verify the consistency of differential expression levels between sepsis and control samples. The box plot exhibited substantial and uniform expression of these six genes in both the training and validation sets. They were markedly downregulated in sepsis (Fig. 4c, d, Supplementary Figure S2). As a result, the following genes were identified as hub genes for subsequent analysis: RORA, L3MBTL2, PHC1, RPA1, CHD3, and RANGAP1. The positions of hub genes were subsequently illustrated, indicating PHC1 on chromosome 12, RORA on chromosome 15, PRA1 and CHD3 on chromosome 17, and L3MBTL2 and RANGAP1 on chromosome 22 (Fig. 4e). Additionally, a GGI network was established to forecast the potential interacting genes or enrichment pathways related to these six hub genes. A strong interaction was observed between L3MBTL2 and MBTD1, associated with histone binding (Fig. 4f).

### Involvement of hub genes in sepsis-associated pathways

**Fig. 5:**
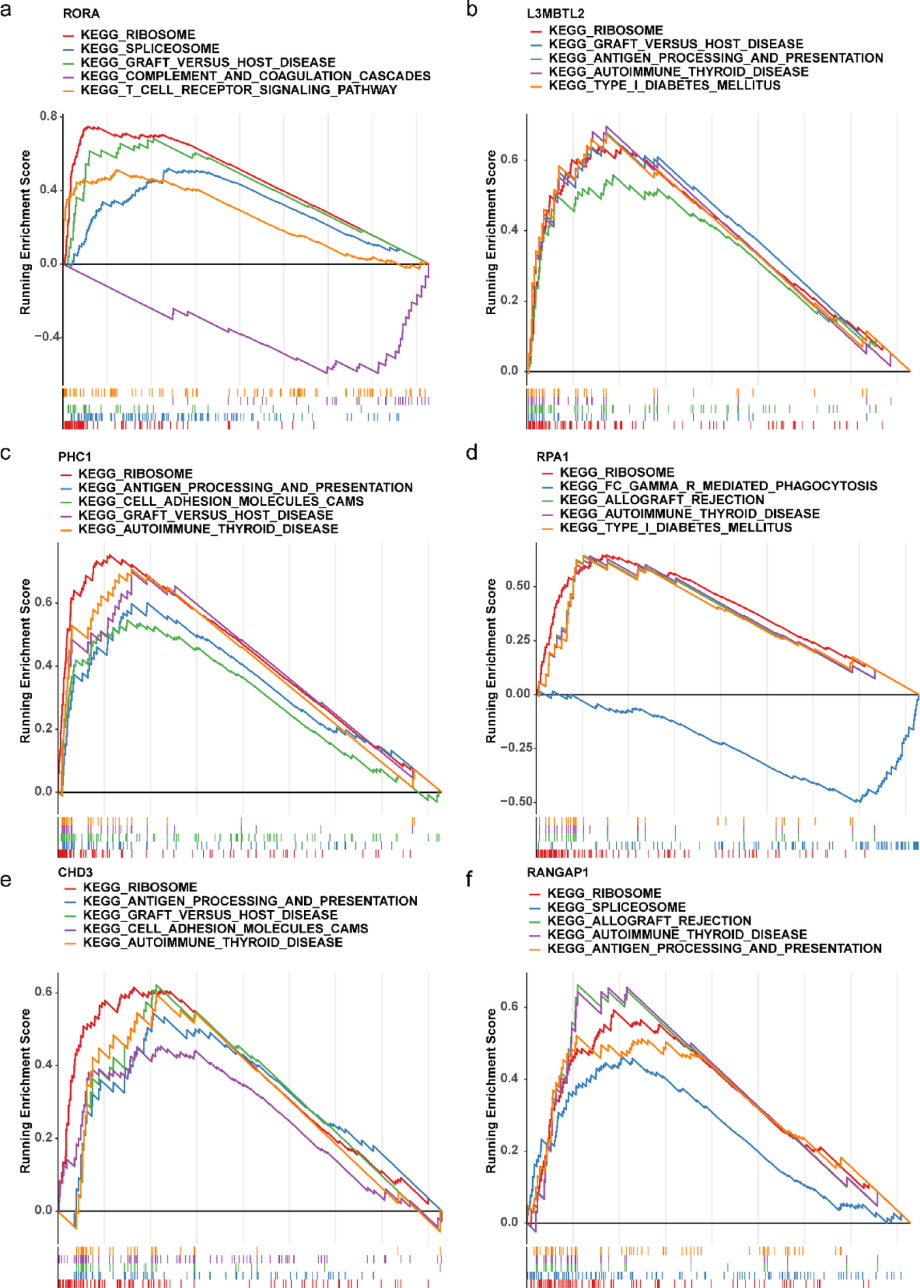
GSEA analysis of hub genes. (**a)** GSEA results associated with RORA; (**b)** L3MBTL2, (**c)** PHC1, (**d)** RPA1, (**e)** CDH3, and (**f)** RANGAP1 within the sepsis.

GSEA was conducted to investigate the potential pathways of the hub genes associated with the pathogenesis of sepsis. Notably, these six hub genes exhibited correlations with ribosome, autoimmune thyroid disease, and spliceosome pathways (Fig. 5a–f).

### Immune cell composition and correlation analysis in sepsis

**Fig. 6:**
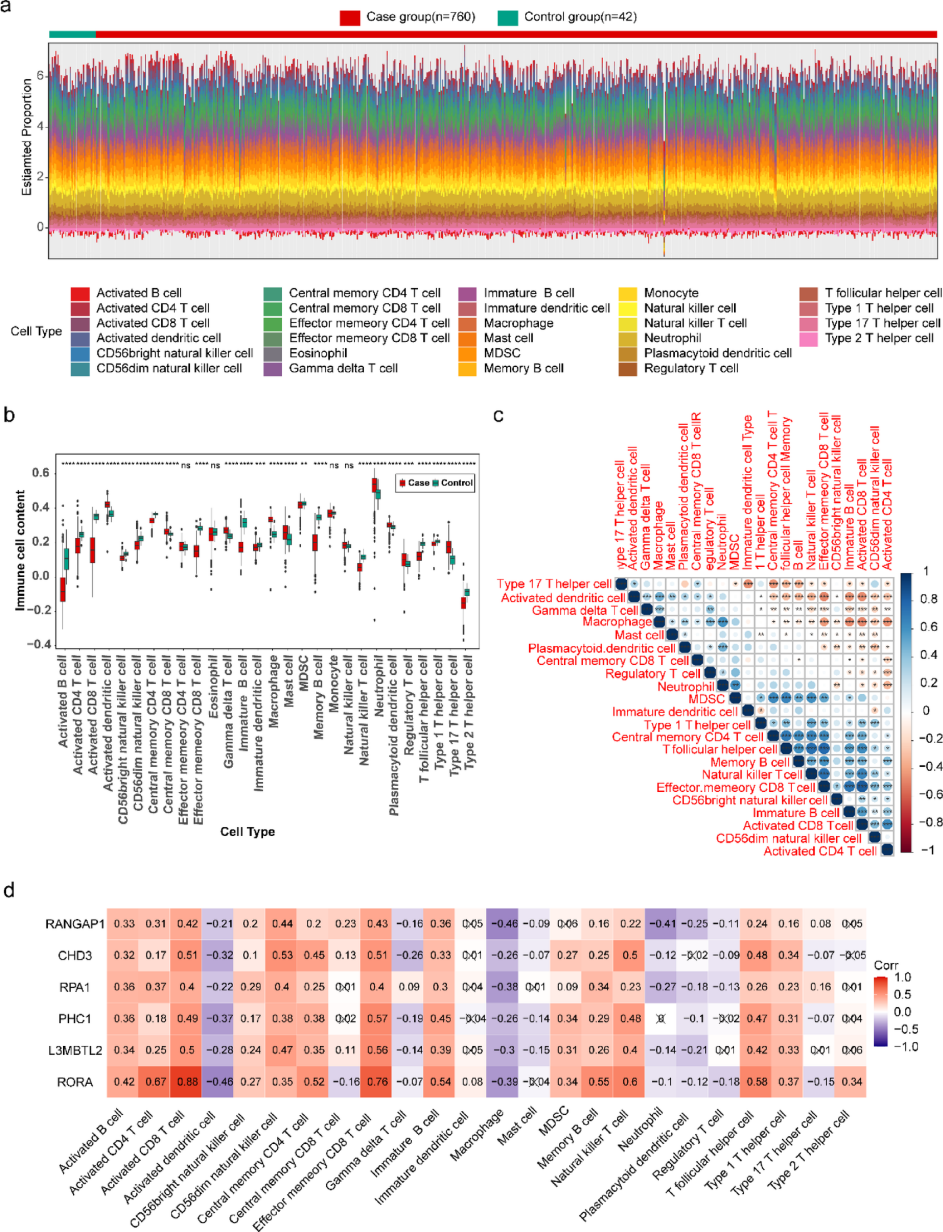
Immune cell composition and correlation analysis in training set. (**a)** Proportions of immune infiltrating cells. (**b)** Boxplot illustrating intergroup differences in immune infiltrating cell composition. (**c)** Heatmap showing the correlation of differentially expressed immune infiltrating cells. (**d)** Heatmap depicting the correlation between key genes and differentially expressed immune infiltrating cells.

The stack diagram illustrated the distribution of 28 immune cell types across all samples in the training set (Fig. 6a). Moreover, the proportions of the remaining 24 immune cells, excluding effector memory CD4^+^ T cells, eosinophils, monocytes, and natural killer cells, varied between the sepsis and control groups (Fig. 6b). Correlation analysis of differentially infiltrating immune cells subsequently demonstrated a significant positive correlation between effector memory CD8 + T cells and activated CD8 + T cells (Fig. 6c). Activated CD8 + T cells exhibited the strongest positive correlation with RORA (*cor* = 0.88), whereas the most pronounced negative correlation was noted between RORA and activated dendritic cells, as well as between RANGAP1 and macrophages (*cor* = − 0.46) (Fig. 6d).

### Subcellular localization analysis of hub genes in sepsis

**Fig. 7:**
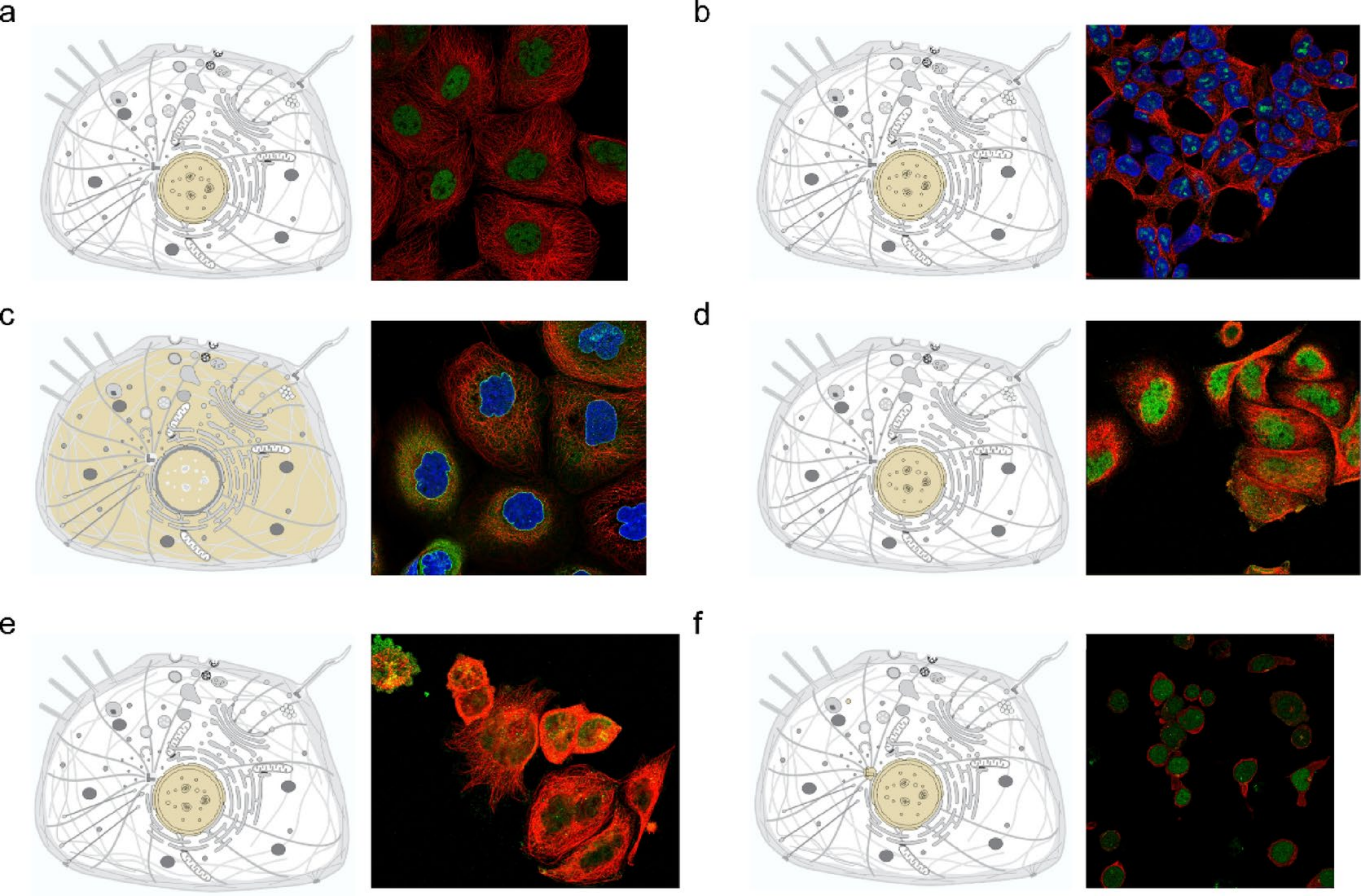
Subcellular localization analysis of hub genes. (**a)** Subcellular mapping of the RORA. (**b)** Subcellular mapping of the L3MBTL2. (**c)** Subcellular mapping of the PHC1. (**d)** Subcellular mapping of the RPA1. (**e)** Subcellular mapping of the CHD3. (**f)** Subcellular mapping of the RANGAP1. Images from UniProt database (https://www.uniprot.org/) and Human Protein Atlas (HPA, https://www.Proteinatlas.org/).

An analysis of subcellular localization was conducted to identify the hub genes and forecast their functional sites. These genes displayed diverse subcellular localizations, predominantly localizing to the nucleus. Furthermore, RPA1 and CHD3 were localized in distinct subcellular structures, specifically the PML body (Fig. 7a and f, Supplementary Table 1).

### Regulatory network and potential therapeutic agents in sepsis

**Fig. 8:**
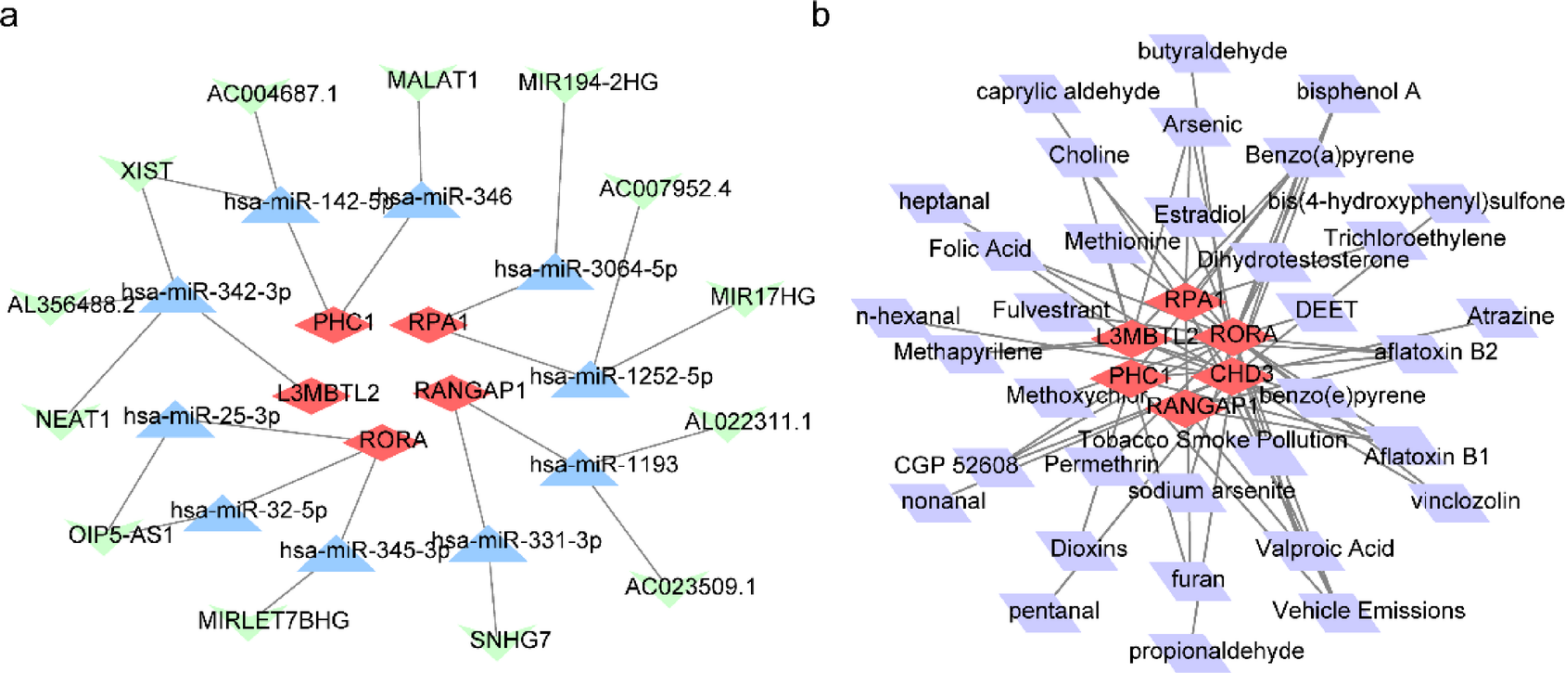
Regulatory network and potential therapeutic agents in sepsis. (**a)** lncRNA-miRNA-mRNA network. (**b)** Interaction network between drugs and hub genes.

To investigate the regulatory mechanism, the miRNAs and lncRNAs associated with the regulated mRNA were predicted. A lncRNA-miRNA-mRNA network comprising 5 mRNAs, 10 miRNAs, and 13 lncRNAs was established, wherein AC004687.1 and MALAT1 modulated PHC1 expression via hsa-miR- 142 - 5p and hsa-miR- 346, respectively (Fig. 8a). Aflatoxin B1 was identified as a prospective therapeutic agent for sepsis based on CHD3, L3MBTL2, PHC1, RANGAP1, and RORA (Fig. 8b).

## Discussion

Sepsis is a critical infectious condition resulting from abnormal reactions of a patient’s immune system. It is characterized by chronic infection and an unregulated systemic inflammatory responses, resulting in tissue damage and ultimately multi-organ failure. SUMOylation modulates the pathogenesis of sepsis by influencing inflammatory responses and immune cell functionality^8,9^. Consequently, it is essential to clarify the pathogenesis of sepsis and establish a theoretical foundation for novel therapeutics.

The convergence of feature genes examined through LASSO regression and the SVM-RFE algorithm showed RORA, L3MBTL2, RPA1, CHD3, and RANGAP1 to harbor a robust diagnostic efficacy with consistent and significant expression patterns across both datasets. Consequently, they were designated as key genes. Previous studies has primarily focused on inflammatory mediators such as TNF-α, IL- 6, and IL- 10, as well as immune cell subsets like T cells and macrophages in the context of sepsis^28,29^. While these studies offer crucial insights into the inflammatory aspect of sepsis, they often overlook the broader regulatory roles of genes involved in processes like SUMOylation, circadian rhythms, and DNA metabolism. Our identification of RORA as a regulator of circadian cycles^30^connects to circadian biology in immune responses, an area that has garnered increasing attention, a healthy circadian cycle may influence the severity, treatment response, and survival outcomes of sepsis^31^, though the depth of analysis linking circadian disruption specifically to sepsis survival remains limited. Furthermore, previous studies have experimentally validated RORA as a potential key regulator in sepsis, as well as an immune dysregulation-related gene^32,33^.

L3MBTL2 exhibits exclusive expression of paternal alleles in multiple hematopoietic progenitors and several differentiated hematopoietic cells subtypes^34^. L3MBTL2 is associated with transcriptional repression, consequently, SUMOylation of L3MBTL2 promotes monoubiquitination or inhibits the deubiquitination of histone H2 AK119^35^. Furthermore, in the study by Huang et al., it was demonstrated that L3MBTL2 inhibits downstream CGA and regulates autophagy to promote pancreatic cancer progression through an H2 AK119ub1-dependent mechanism^36^. Although no research has directly linked L3MBTL2 to sepsis, numerous studies have highlighted its unique oncogenic role in tumor development^37,38^. RPA1 is a nucleoprotein complex that governs DNA metabolism, participating in DNA replication, repair, recombination, and telomere maintenance. It orchestrates cellular responses to DNA damage by activating ataxic telangiectasia and RAD3- related protein (ATR) kinases^39^. In addition, RPA1 governs T cell homeostasis and host inflammatory response^40^. Moreover, in various diseases, RPA1 exerts its effects by regulating chromatin structure^41^. RANGAP1 associates with the nuclear pore complex and is involved in the regulation of nuclear transport. Notably, RANGAP1 knockdown has been observed to induces lymphoma cell mortality and cell cycle arrest^42^. Although studies have suggested that circadian disruptions can influence the severity of infections and immune responses^43^, our study specifically points to the role of circadian regulators like RORA in modulating the inflammatory response in sepsis. This relationship could explain why patients with advanced sepsis exhibit altered immune functions and outcomes. L3MBTL2, RPA1, and RANGAP1 have not been previously associated with sepsis, offering new avenues to explore their roles. While previous studies have highlighted the importance of SUMOylation in immune responses and its role in regulating inflammation-related protein interactions^44^, our findings further extend this knowledge by specifically linking SUMOylation to key genes that influence immune cell functions and inflammatory pathways in sepsis. Herein, the GO and KEGG enrichment analysis of the eight candidate genes revealed four significantly enriched signaling pathways: Nucleocytoplasmic transport, polycomb repressive complex, mismatch repair, and circadian rhythm. These pathways are potentially involved in sepsis, particularly sleep and circadian rhythm disorders. Importantly, circadian rhythm disorders have been significantly associated with the advancement of sepsis-induced cardiomyopathy, which is a primary cause of mortality^45^. Moreover, the biological clock has also been associated with immune dysfunction and sepsis^46^, as well as diurnal variations in mortality in animal sepsis models^31,47^.

We observed that advanced sepsis leads to CD8 + T cell apoptosis and lymphocytopenia, consistent with earlier findings^48^.Immunosuppression induced by sepsis is characterized by lymphocytopenia and loss of immune function^49^, while T lymphocytes are key in anti-infection immunity. In sepsis, the number of B cells, CD4 + T and CD8 + T cells can be significantly reduced^50^. Dendritic cells are the major antigen presenting cells and modifies innate and adaptive immune responses. In sepsis, the number of dendritic cells is significantly reduced, resulting in inhibited antigen presentation, abnormal cytokine secretion, and impaired T cell activation. Importantly, disrupted SUMOylation can affect the development or function of T cells. T cell proliferation and activation are regulated by SUMOylation of numerous transcription factors, including NFAT (nuclear factor of activated T cells)^51^. The high expression of SUMO is likely to affect the development and maturation of dendritic cells^10^. However, dendritic cell maturation is typically not influenced by SUMO2 overexpression; nonetheless, naïve CD4^+^T cells are converted to TH2-type in vitro^52^. Furthermore, inflammation is regulated by SUMO in macrophages while SUMO2 has been known to influence the antiviral response of dendritic cells^53^.

Nevertheless, the reduction of organ damage in sepsis and the enhancement of survival can be achieved by inhibiting macrophage-mediated inflammation and apoptosis^54,55^. The specific role of SUMOylation in this context remains underexplored, investigating how epigenetic modifications like SUMOylation influence the survival and function of these immune cells could provide insight into therapeutic targets for enhancing immune resilience in sepsis.

NF-κB also comprises a critical modulator of the immune response. Upon activation of NF-κB, the secretion of an abundance of inflammatory cytokines exacerbate secondary damage and induce apoptosis in septic cells^56,57^. It is worth noting that the NF-κB pathway induces inflammation and the secretion of chemokines through SUMOylation^58^. Although previous studies have established the relationship between NF-κB signaling and immune responses in sepsis, our findings suggest that SUMOylation may modulate these pathways in a more subtle manner, potentially influencing the survival and activation of immune cells.

The identification of novel key genes and their pathways offers promising avenues for the development of targeted therapies in sepsis. Our study suggests possible biomarkers for diagnosing sepsis and monitoring therapeutic responses, particularly in relation to immune system performance and circadian health. Targeting RORA to restore circadian rhythm could be an innovative therapeutic approach to mitigate immune dysfunction and improve patient outcomes. In summary, our study utilized the publicly available GEO database to investigate SUMOylation in sepsis and decode a valuable genetic signature for the diagnosis and therapy of sepsis. However, our study has certain limitations. First, the methodologies employed, including WGCNA, LASSO, and SVM-RFE, are traditional approaches that rely heavily on a single type of data or conventional feature selection methods. Second, there is a lack of sufficient proteomics and metabolomics data. Finally, there is a deficiency of adequate experimental validation. Therefore, in future research, advanced multi-omics integration tools such as MOGONET or M3 NetFlow should be considered. These tools are capable of more effectively integrating different types of data, including genomics, transcriptomics, proteomics, and metabolomics, thereby enabling a better capture of potential interactions between data types.Additionally, it is crucial to expand the sample collection in subsequent studies to ensure the acquisition of sufficient proteomics and metabolomics samples. Lastly, in vitro experiments, such as cell culture studies, should be conducted at the cellular level, followed by in vivo validation in animal models. By applying advanced multi-omics integration tools, improving data sample collection, and conducting comprehensive experimental validation, the overall study will become more logically rigorous and complete.

## Conclusions

This study shows that hub genes (RORA, L3MBTL2, PHC1, RPA1, CHD3, and RANGAP1) may distinguish controls and diseases to facilitate diagnosis of sepsis. Further studies are needed to validate our findings and to elucidate their therapeutic implications in the context of sepsis.

## Electronic supplementary material

Below is the link to the electronic supplementary material.

Supplementary Material 1

## Data Availability

The datasets extracted and/or analysed during the current study are available in the GEO database repository, [https://www.ncbi.nlm.nih.gov/gds for GSE65682, GSE95233, GSE28750, GSE134347] and MSigDB for SUMO-RGs (searched with “sumoylation” in https://www.gsea-msigdb.org/gsea/msigdb).

## Abbreviations

SUMOylation: Small ubiquitin-like modifier mediated modification
WGCNA: Weighted Gene Co-expression Network Analysis
DEGs: Differentially expressed genes
SUMO-RGs: SUMOylation-related genes
LASSO: Least Absolute Shrinkage and Selection Operator
SVM-RFE: Support Vector Machine-Recursive Feature Elimination
AUC: Area under the curve
SUMOylation: Small ubiquitin-like modifier
GEO: Gene Expression Omnibus
GO: Gene Ontology
KEGG: Kyoto Encyclopedia of Genes and Genomes
PPI: Protein-protein interaction
ROC: Receiver operating characteristic
GSEA: Gene Set Enrichment Analysis
GGI: Gene-gene interaction
RORA: Related orphan receptor A
L3MBTL2: Lethal (3) malignant brain tumor-like protein 2
RPA1: Replication protein A1
CHD3: Chromodomain-helicase-DNA-binding protein 3
RANGAP1: RanGTPase activating protein 1
