# Supplementary material for: Integrating bioinformatics and machine learning to discover sumoylation associated signatures in sepsis
Source: Sci Rep. 2025 Apr 24;15:14398. doi: 10.1038/s41598-025-96956-x (PMC12022290; doi:10.1038/s41598-025-96956-x)
Supplement: Supplementary file 1 — Supplementary Material 1 [file 41598_2025_96956_MOESM1_ESM.pdf]

Supplementary Information

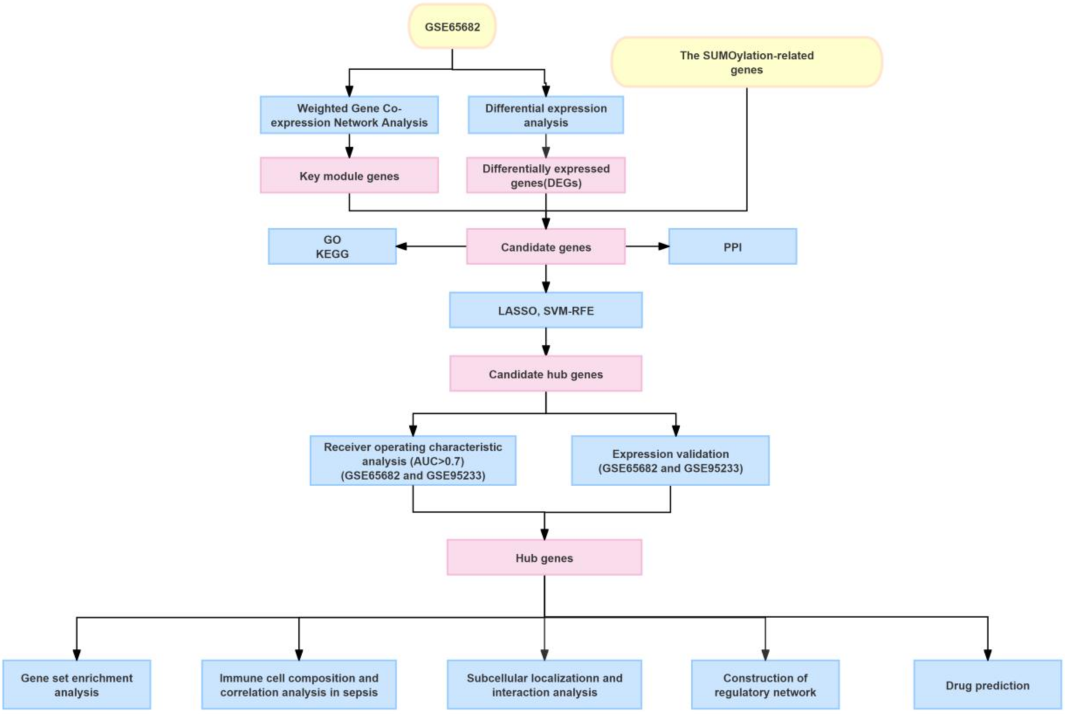

Supplementary Figure S1 Flow chart of the research program.

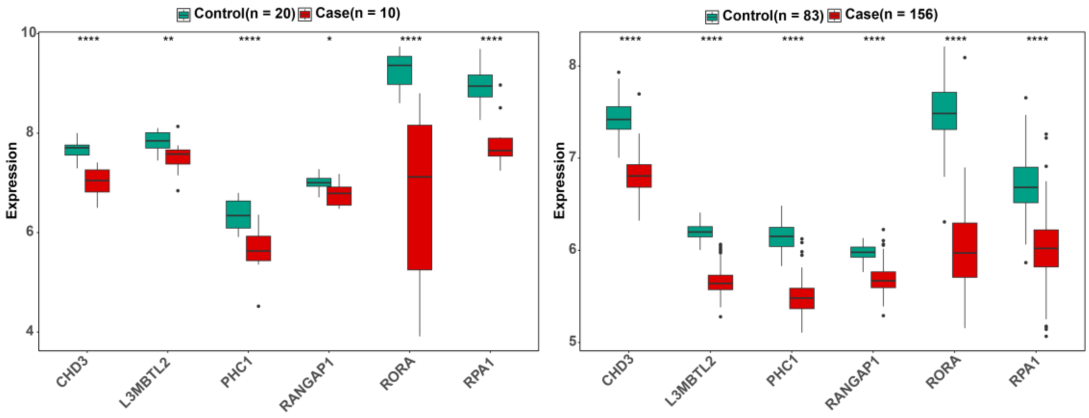

Supplementary Figure S2 Core genes expression in validation set GSE28750. left plot was GSE28750 dataset, right plot was GSE134347.

Gene	Degree
RORA	0
L3MBTL2	2
PHC1	2
RPA1	0
CHD3	1
RANGAP1	2

Supplementary Table S1 The degree values of these 6 genes.
